# Machine-learning algorithm to predict home delivery after antenatal care visit among reproductive age women in East Africa

**DOI:** 10.3389/fgwh.2025.1461475

**Published:** 2025-06-05

**Authors:** Agmasie Damtew Walle, Shimels Derso Kebede, Jibril Bashir Adem, Daniel Niguse Mamo

**Affiliations:** ^1^ Department of Health Informatics, School of Public Health, Asrat Woldeyes Health Science Campus, Debre Berhan University, Debre Birhan, Ethiopia; ^2^ Department of Health Informatics, School of Public Health, College of Medicine and Health Sciences, Wollo University, Dessie, Ethiopia; ^3^ Department of Public Health, College of Medicine and Health Sciences, Arsi University, Asella, Ethiopia; ^4^ Department of Health Informatics, College of Medicine and Health Sciences, Arba Minch University, Arba Minch, Ethiopia

**Keywords:** machine learning, home delivery, ANC visit, East Africa, prediction

## Abstract

**Background:**

Maternal and child health remains a global public health issue, particularly in low- and middle-income countries where maternal and child mortality are extremely high. The World Health Organization estimates that close to 287,000 women die annually due to pregnancy and childbirth complications, and the majority of these deaths occur where skilled birth attendants are not readily available. Reducing the prevalence of home delivery is a key strategy for lowering the maternal mortality rate. Although several studies have explored home delivery and antenatal care (ANC) utilization independently, limited evidence exists on predicting home delivery after ANC visits using machine-learning approaches in East Africa.

**Methods:**

This study utilized a community-based, cross-sectional design with data from the most recent Demographic and Health Surveys conducted between 2011 and 2021 in 12 countries in East Africa countries. A total weighted sample of 44,123 women was analyzed using Python version 3.11. Nine supervised machine-learning algorithms were applied, following Yufeng Guo's steps for supervised learning. The random forest (RF) model, selected as the best-performing algorithm, was used to predict home delivery after ANC visits. A SHapley Additive exPlanations analysis was conducted to identify key predictors influencing home delivery decisions.

**Results:**

Home delivery after ANC visits was most prevalent in Malawi (17.88%), Uganda (15.38%), and Kenya (11.3%), and was low in Comoros (2.38%). Living in rural areas and late ANC initiation (second trimester) increased the likelihood of home delivery. In contrast, factors such as higher household income, husband’s level of primary and secondary education, contraceptive use, shorter birth intervals, absence of distance-related barriers to healthcare, and attending more than four ANC visits were associated with a lower likelihood of home delivery.

**Conclusion:**

The study demonstrates that home delivery after ANC visits was high. The RF machine-learning algorithm effectively predicts home delivery. To reduce home deliveries, efforts should improve early ANC initiation, enhance healthcare quality, and expand facility-based services. Policymakers should prioritize increasing health facility accessibility, promoting media-based health education, and addressing financial barriers for women with low incomes. Strengthening these areas is crucial for improving maternal and neonatal health outcomes in East Africa.

## Introduction

A home birth after an antenatal care (ANC) visit is described as a delivery that occurs at home without the presence of a skilled birth attendant, despite the mother having received ANC care (1). Maternal health remains a top priority on the global health agenda (2). Every minute, women around the world die due to problems related to pregnancy and childbirth, most of which are preventable (3). In 2017 alone, approximately 295,000 women died from pregnancy- and childbirth-related issues (3).

Maternal and child mortality remain major global health challenges, with sub-Saharan Africa accounting for 70% of global maternal deaths. In East Africa, home delivery remains prevalent, particularly in Malawi, Uganda, and Kenya, due to rural residence, cultural practices, and limited access to healthcare (3). Despite efforts to enhance ANC utilization, many women still deliver at home, increasing health risks to them and their newborns (4).

Pregnancy- and childbirth-related complications tend to be the reason for the deaths and disabilities that occur worldwide. The main reasons for death include infection, prolonged or obstructed labor, complications from a surgical abortion, hemorrhage, malaria during pregnancy, and anemia (5). The use of professional care during childbirth remains significantly lower in sub-Saharan Africa and South/Southeast Asia, with the exception of a few countries such as Benin, Namibia, Zimbabwe, and Vietnam. In Ethiopia, home delivery is especially prevalent in the Afar and Somali regions, where 89.5% and 81.7% of women, respectively, gave birth at home, compared with just 3.3% of women living in Addis Ababa (6).

Studies have identified a range of statistically significant predictors of home delivery after an ANC visit. These include the women’s educational status (7–13), cultural factors (8, 9, 13, 14), geographic region (7), parity (7), limited access to healthcare facilities (8, 9), poor quality of care (8, 9), lack of transportation (4, 8, 9), maternal age (6, 11, 12, 14, 15), marital status (4), environment (13, 16), distance to health facilities (3, 4, 7, 9, 10, 17), source of health information (3, 4, 7, 10), antenatal care visits (3, 7–9, 18), birth order (7, 8), household wealth index (7, 8, 11, 19), place of residence (3, 6, 7, 9, 12, 18), religion (7, 8, 14), employment status of the woman and her husband (6), knowledge about place of delivery (3, 11, 12, 18), unplanned pregnancy (10), and decision-making regarding the place of delivery (3, 11, 13).

Home delivery poses significant health risks, increasing the likelihood of pregnancy-related complications and neonatal illness or death (20). Studies have shown that perinatal mortality rates are 21% higher for home births compared with deliveries in healthcare facilities, and home births are associated with infections and other adverse outcomes for both mothers and newborns (10).

Home delivery after an ANC visit remains a major public health concern. Reducing the prevalence of home deliveries is one of the most effective ways for lowering maternal mortality rates (3). This study is important when prioritizing the establishment of public health measures because it uses weighted pooled DHS data from 12 countries in East Africa, providing adequate power to accurately detect predictors of home delivery after ANC visits. Existing studies on home delivery rely on traditional statistical methods, which often fail to capture complex interactions among determinants. However, this study fills the gap by using machine learning (ML) to enhance predictive accuracy. A SHapley Additive exPlanations (SHAP) analysis is applied to identify the most influential predictors of women's delivery choices. This research is significant for African policymakers and global health researchers as it enhances predictive modeling for maternal health, provides data-driven insights for targeted interventions, and supports evidence-based policies to improve institutional deliveries (7). Therefore, the aim of this study was to predict home delivery after ANC visits among women of reproductive age in East Africa using supervised machine-learning algorithms.

## Methods and materials

### Study design and study setting

A community-based cross-sectional study was conducted to predict home delivery after ANC visits among women aged 15–49 years using recent DHS data from East African countries, collected between 2011 and 2021. East Africa comprises 19 countries: Rwanda, Seychelles, Somalia, Tanzania, Uganda, Zambia, South Sudan, Zimbabwe, Burundi, Comoros, Djibouti, Ethiopia, Eritrea, Kenya, Madagascar, Malawi, Mauritius, Mozambique, and Sudan (21). However, only 14 of these countries have DHS data available, whereas five countries (Djibouti, Somalia, South Sudan, Seychelles, and Mauritius) lack DHS datasets. Among the 14 countries with data, Eritrea has restricted DHS data and Sudan’s dataset dates back to 1989–1990. Thus, this study included 12 countries with recent publicly available DHS datasets (Burundi, Ethiopia, Comoros, Uganda, Rwanda, Tanzania, Mozambique, Madagascar, Zimbabwe, Kenya, Zambia, and Malawi).

Enumeration areas (EA) were selected independently within each sampling stratum using probability sampling proportional to EA size. Households were then systematically selected from each chosen EA. The DHS report included a comprehensive description of the sampling methodology (22, 23).

### Data source and sampling technique

The study was conducted using publicly available datasets based on the Demographic and Health Surveys (DHS). Different datasets, such as those for men, women, children, births, individuals, and households, are included in each country's survey; for this study, we used the Individual Record (IR) file. DHS used the Population and Housing Census (PHC) as a sampling frame for a two-stage stratified cluster sampling technique.

### Populations

We extracted the data of 75,047 women of reproductive age in the study. However, after going through the data (excluding women who did not have an ANC visit, missing values, and unknown responses), a total weighted sample of 44,123 respondents was included in the study for further analysis.

### Study variables

The outcome variable, home delivery after ANC visit, is defined as a woman who gives birth at home despite having attended at least one ANC visit during her pregnancy. It was measured as a binary variable (1 = home delivery, 0 = facility-based delivery) (24). Independent variables for this study included maternal age, number of ANC visits, area of residence, birth interval, level of education of the mother and her husband, marital status, household wealth index, media exposure, sex of household head, previous contraceptive use, the timing of ANC visits, and health facility issues (Supplementary Table S1).

### Data management and analysis

As recommended by the DHS, the data were weighted using the primary sampling unit, sampling weight, and strata to restore representativeness and account for the complex sampling design, ensuring accurate statistical analysis. Sampling statisticians determined the appropriate sample size for each stratum.

A total of 75,047 actual samples with selected variables were extracted using STATA software version 17 and exported as a CSV file. Further analysis was conducted in Jupyter Notebook version 3.11. Preprocessing steps included explanatory data analysis, missing value management, data discretization, outlier detection, target feature balancing, and feature selection. Finally, the dataset was split into training (80%) and test (20%) subsets, and only features that passed the selection process were used for modeling.

This study used the following nine supervised machine-learning algorithms to predict home delivery among East African women of reproductive age after an ANC visit: Support Vector Machine (SVM), Ada Boost (AdB), Gaussian naïve Bayes (GNB), Multi-Layer Perception (MLP), Decision Tree (DT), Logistic Regression (LR), K Nearest Neighbors (KNN), Extreme Gradient Boosting (XG Boost), and Random Forest (RF) (25–28).

Models training used tenfold cross-validation. A total of 12 recent DHS datasets from East African countries were analyzed. Performance was evaluated using accuracy, precision, recall, F1-score, and area under the curve (AUC) to identify the best-performing model, whose hyperparameters were subsequently adjusted. To identify the best-performing model, we followed a systematic approach that included model evaluation, comparison, and selection based on the metrics. In statistical analyses, packages were applied, including imblearn (29) to handle imbalanced datasets through resampling techniques, sklearn (30) for implementing machine-learning algorithms and model evaluation, and SHAP (31) to interpret model predictions by explaining the contribution of each feature.

SHAP was used to determine feature importance, offering insights into how each variable influenced the prediction of home delivery after an ANC visit. Numerous studies have used SHAP as a feature selection method, demonstrating that incorporating SHAP values into machine-learning models works better in terms of classification performance and model explainability. By plotting the total Shapley value of each feature, it is easier to understand the influence of individual predictors on the likelihood of home delivery after an ANC visit (27, 32).

**Figure 1:**
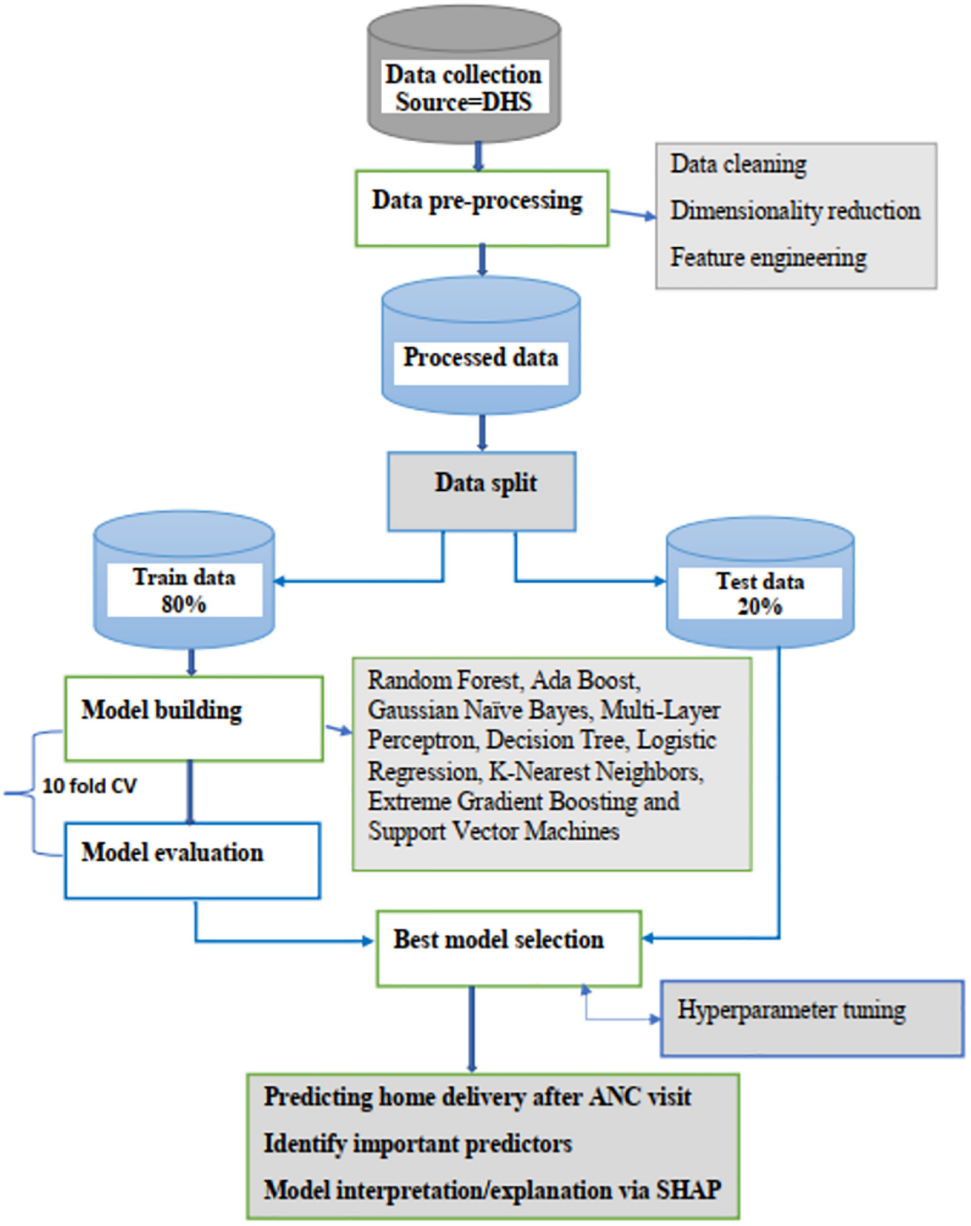
Overview flow chart of methodologies.

In addition, the contributions of each feature to the prediction of the positive class (home delivery) were explained using a waterfall plot (27). In this plot, the *x*-axis represents the probability that a sample will be assigned to the “home delivery” class, while the *y*-axis lists the independent variables and their corresponding values for each sample. Each horizontal bar reflects each feature's contribution. Positive contributions (red bars) indicate that the feature increases the probability of home delivery, whereas negative contributions (blue bar) suggest a decreased likelihood of this outcome. The complete workflow methodology is presented in Figure 1.

## Results

### Characteristics of the study respondents

The mean age of the participants was 28.41 years. Of the study participants, 33,048 (74.9%) were from rural areas. In total, 7,889 (17.88%) were from Malawi and 2.38% were from Comoros. Of the participants, 16,693 (37.83%) had attained primary education, 19,147 (43.40%) had a low wealth index, 28,779 (65.22%) were married, 29,472 (66.80) were exposed to media, and 31,713 (71.87%) had attended two to four ANC visits. Of the participants, 26,268 (59.53%) had no issues with the distance to health facilities (Table 1).

**Table 1:** Sociodemographic characteristics of reproductive-age women in East Africa, DHS 2011–2021.

Variable	Category	Frequency	Percentage
Residence	Urban	11,075	25.1
	Rural	33,048	74.9
Country	Burundi	2,764	6.26
	Ethiopia	3,267	7.40
	Kenya	4,995	11.3
	Comoros	1,052	2.38
	Madagascar	2,753	6.24
	Malawi	7,889	17.88
	Mozambique	1,548	3.51
	Rwanda	4,014	9.10
	Tanzania	3,002	6.80
	Uganda	6,788	15.38
	Zambia	4,394	9.96
	Zimbabwe	1,657	3.76
Maternal age in years	15–24	7,764	17.60
	25–34	23,365	52.95
	35–49	12,994	29.45
Maternal education	No formal education	9,492	21.51
	Primary	16,693	37.83
	Secondary	14,587	33.06
	Higher	3,351	7.60
Marital status	Single	11,778	26.70
	Married	28,779	65.22
	Widowed	1,378	3.12
	Divorced	2,188	4.96
Wealth index	Poor	19,147	43.40
	Middle	8,410	19.06
	Rich	16,566	37.54
Media exposure	Yes	29,472	66.80
	No	14,651	33.20
Sex of household head	Male	36,042	81.69
	Female	8,081	18.31
Previous contraceptive use	No	22,523	51.05
	Yes	21,600	48.95
Timing of ANC visit	First trimester	14,174	32.12
	Second trimester	26,340	59.70
	Third trimester	3,609	8.18
Number of ANC visit	One	1,383	3.13
	2–4	31,713	71.87
	Above 4	11,027	25.0
Birth interval	Short	18,286	41.44
	Long	25,837	58.56
Husband education	No formal education	7,939	18.00
	Primarily education	22,284	50.50
	Secondary education	11,005	24.94
Husband education	No formal education	7,939	18.00
	Primarily education	22,284	50.50
	Secondary education	11,005	24.94
	Higher education	2,895	6.56
The distance to a health facility problem	Big problem	17,855	40.47
	Not a big problem	26,268	59.53

## Machine-learning analysis of home delivery after ANC visit

### Data balancing

**Figure 2:**
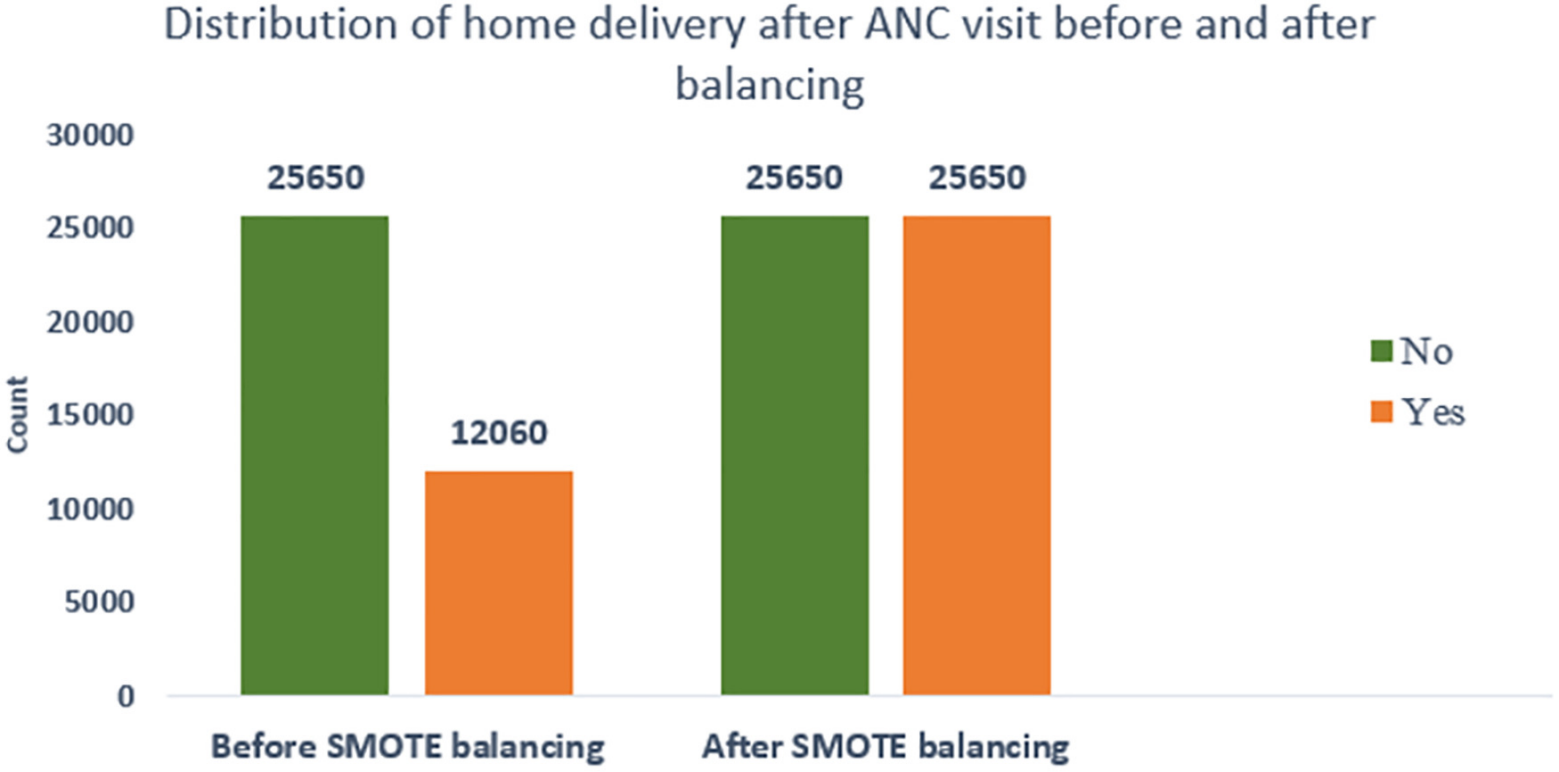
SMOTE balancing of home deliveries after ANC visits among women of reproductive age in East Africa, DHS 2011–2021.

To improve model reliability, synthetic minority oversampling technique (SMOTE) oversampling created 13,590 synthetic observations for the minority class. The total distribution of home deliveries after ANC visits was modified from 12,060 home deliveries and 25,650 non-home deliveries to 25,650 in each class (Figure 2).

### Model performance comparison

Among the classifiers assessed using imbalanced training data with stratified tenfold cross-validation, the Support Vector Machine proved the most effective, achieving an accuracy of 74.82% and an area under the receiver operating characteristic (ROC) curve of 66.17%. However, after applying the SMOTE oversampling technique to balance the training data, the Random Forest model was the best predictive model, with an accuracy of 70.60% and a higher AUC of 77.88% (Table 2).

**Table 2:** Model comparison through cross-validation of training data.

Machine-learning models	Performance	Unbalanced (%)	Balanced (%)
RF	Accuracy	72.11	70.60
	AUC	68.06	77.88
SVM	Accuracy	74.82	68.57
	AUC	66.17	74.41
LR	Accuracy	74.81	67.36
	AUC	73.10	74.18
KNN	Accuracy	71.00	67.67
	AUC	65.38	72.87
AdB	Accuracy	74.72	67.41
	AUC	73.08	74.17
GNB	Accuracy	66.13	64.70
	AUC	69.99	70.95
MLP	Accuracy	74.34	68.82
	AUC	72.08	75.60
DT	Accuracy	74.32	65.69
	AUC	70.62	71.98
XG Boost	Accuracy	74.34	69.35
	AUC	71.92	76.20

**Figure 3:**
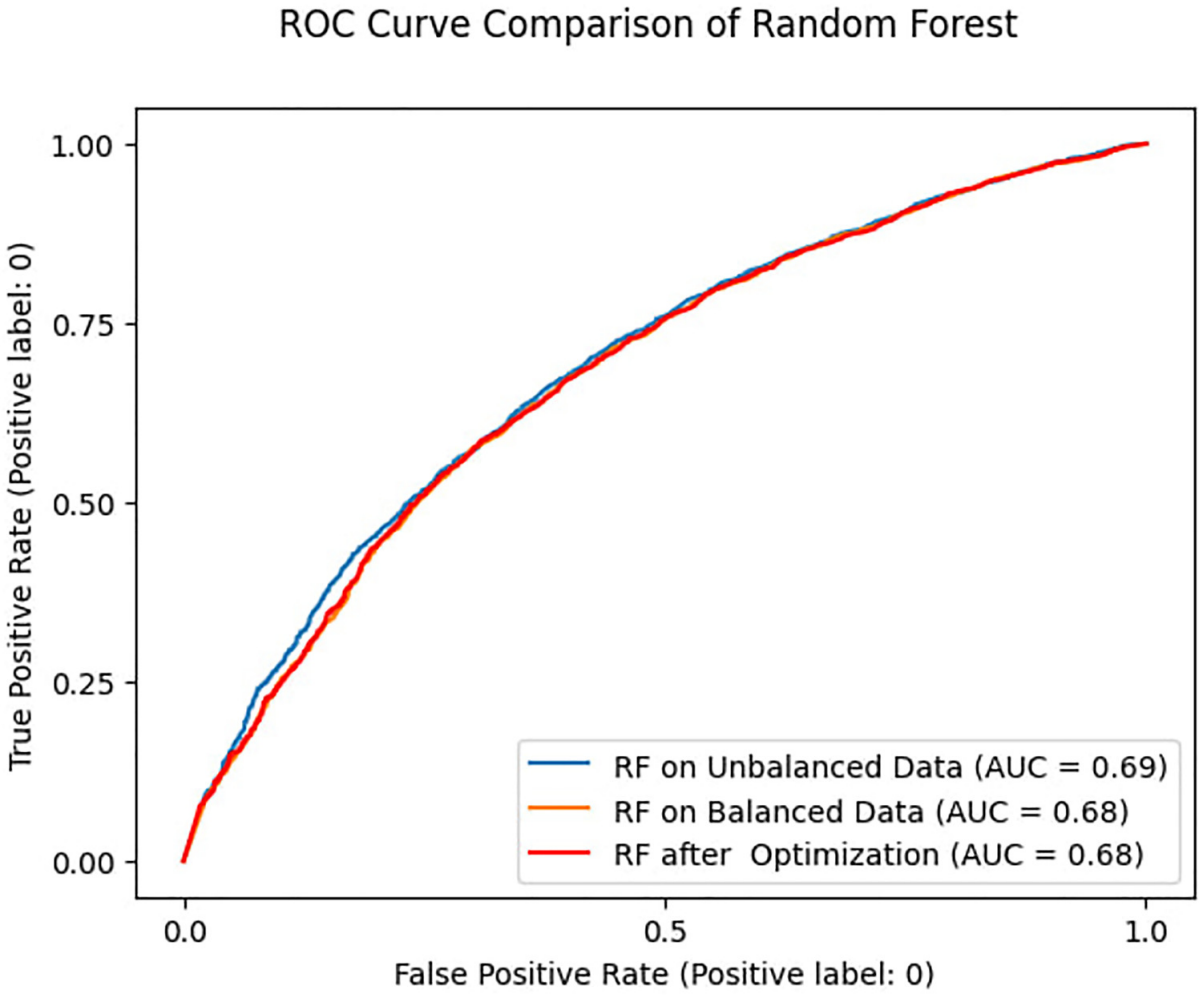
Comparison of Random Forest model predictions.

When tested on unseen data, the Random Forest model produced an AUC of 0.69 and 0.68 for the balanced and unbalanced training datasets, respectively. In addition, after hyperparameter tuning, the Random Forest model predicted an AUC of 0.68 (Figure 3).

### Hyperparameter tuning of RF

A Random Forest model developed with optimized hyperparameters and evaluated using tenfold cross cross-validation on balanced training data achieved an accuracy of 79% and an AUC of 0.78 (Table 3).

**Table 3:** Default and optimally tuned hyperparameters of the random forest model.

Hyperparameter	Default	Optimal value
Number of trees	100	250
Number of features considered for the best split	The square root of the number of features	0.21
The minimum number of samples required to split an internal node	2	2
The minimum number of samples required to be at a leaf node	1	1
Number of samples to draw from X to train each base estimator	None	0.96

### Important feature selection using RF

**Figure 4:**
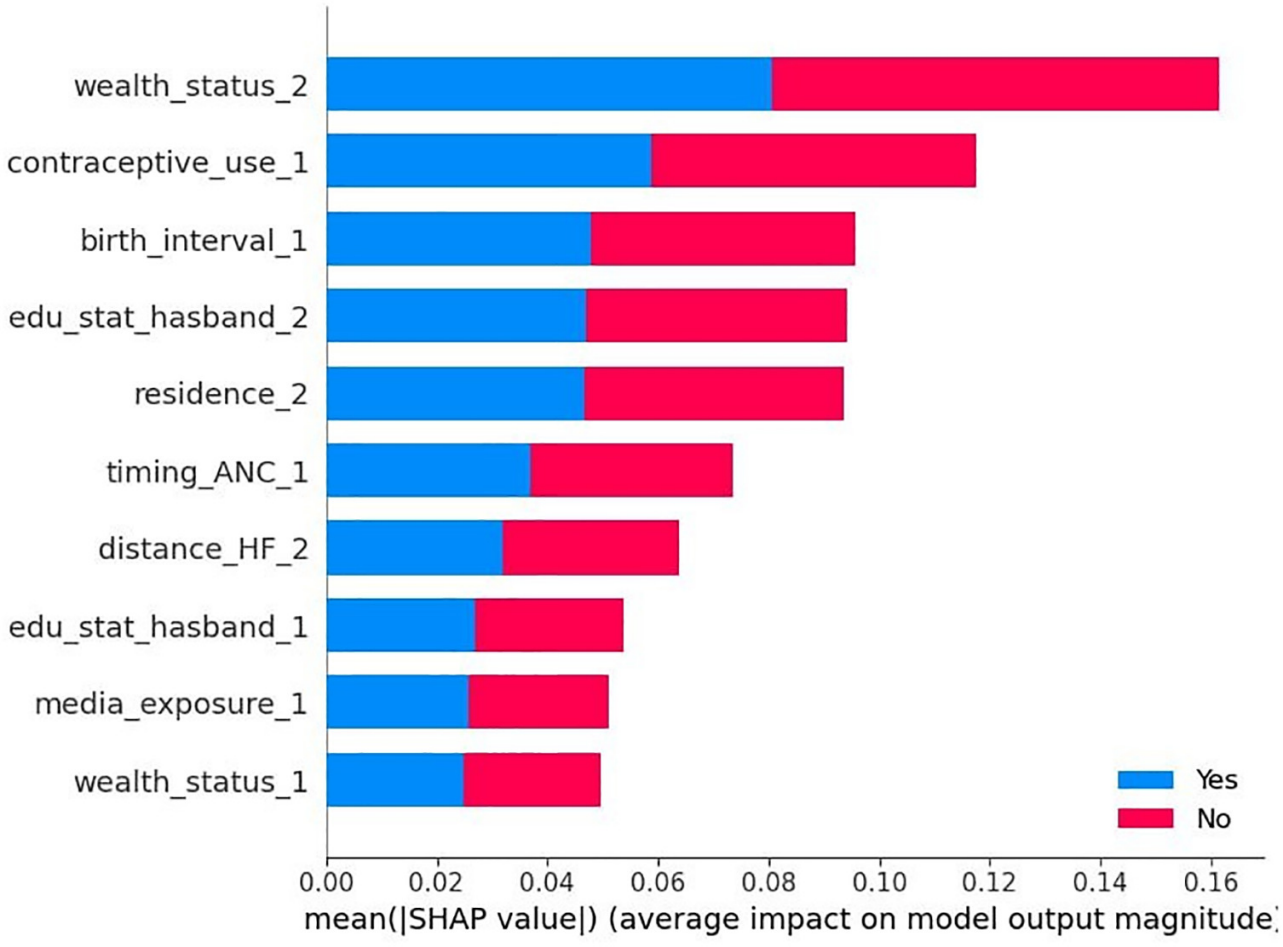
SHAP global importance plot of optimized random forest model.

According to the optimized Random Forest model with test data, the SHAP global importance scores identified the top 10 predictors of home delivery after an ANC visit. These predictors are ranked in descending order according to their mean absolute SHAP values, indicating their influence on the outcome variable. The five most important predictors were: belonging to a rich household (wealth_status = 2), contraceptive use (contraceptive_use = 1), short birth interval (birth_interval = 1), husband’s level of secondary education (edu_status_husband = 2), and residing in rural areas (residence = 2). Other significant predictors included ANC in the second trimester (timing_ANC = 1), difficulty with distance to a healthcare facility (distance_HF = 2), husband’s level of primary education (edu_status_husband = 1), media exposure (media_exposure = 1), and middle household income (wealth_status = 1). As shown in Figure 4, the red and blue colors occupy half of the horizontal rectangles for each class, indicating that each variable has an equal impact on both home delivery after ANC visit (label = yes) and institutional delivery after ANC visit (label = no) outcomes.

### Model interpretation/explanation

**Figure 5:**
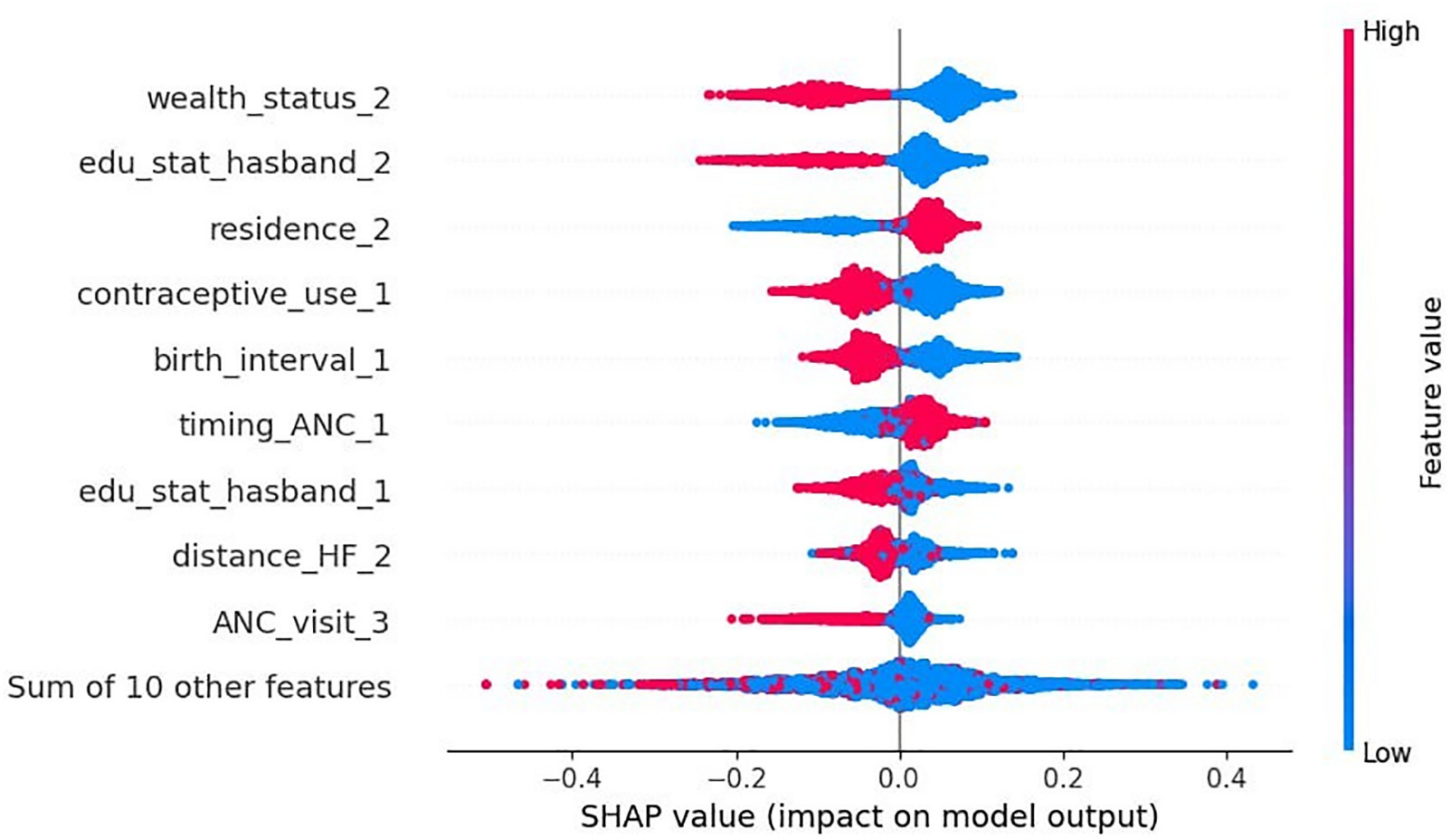
Beeswarm plot of mean absolute SHAP value via optimized random forest model.

According to the beeswarm plot, the points that are to the right of the vertical line (0 SHAP value) increase the likelihood of home delivery after the ANC visit, while those on the left decrease it. The red line represents higher feature values (coded as 1) and the blue line represents lower values (coded as 0). Accordingly, being a rural resident (residence = 2) and attending ANC visits in the second trimester (timing_ANC = 1) increase the likelihood of a home delivery. In contrast, belonging to a rich household (wealth_status = 2), husband’s level of secondary education (edu_status_husband = 2), contraceptive use (contraceptive_use_1), short birth interval (birth_interval = 1), husband’s level of primary education (edu_status_husband = 1), having no difficulties with distance to health facilities (distance_HF = 2), and attending more than four ANC visits (ANC_visit = 3) were factors that decreased the likelihood of a home delivery in East Africa (Figure 5).

### Waterfall plot

**Figure 6:**
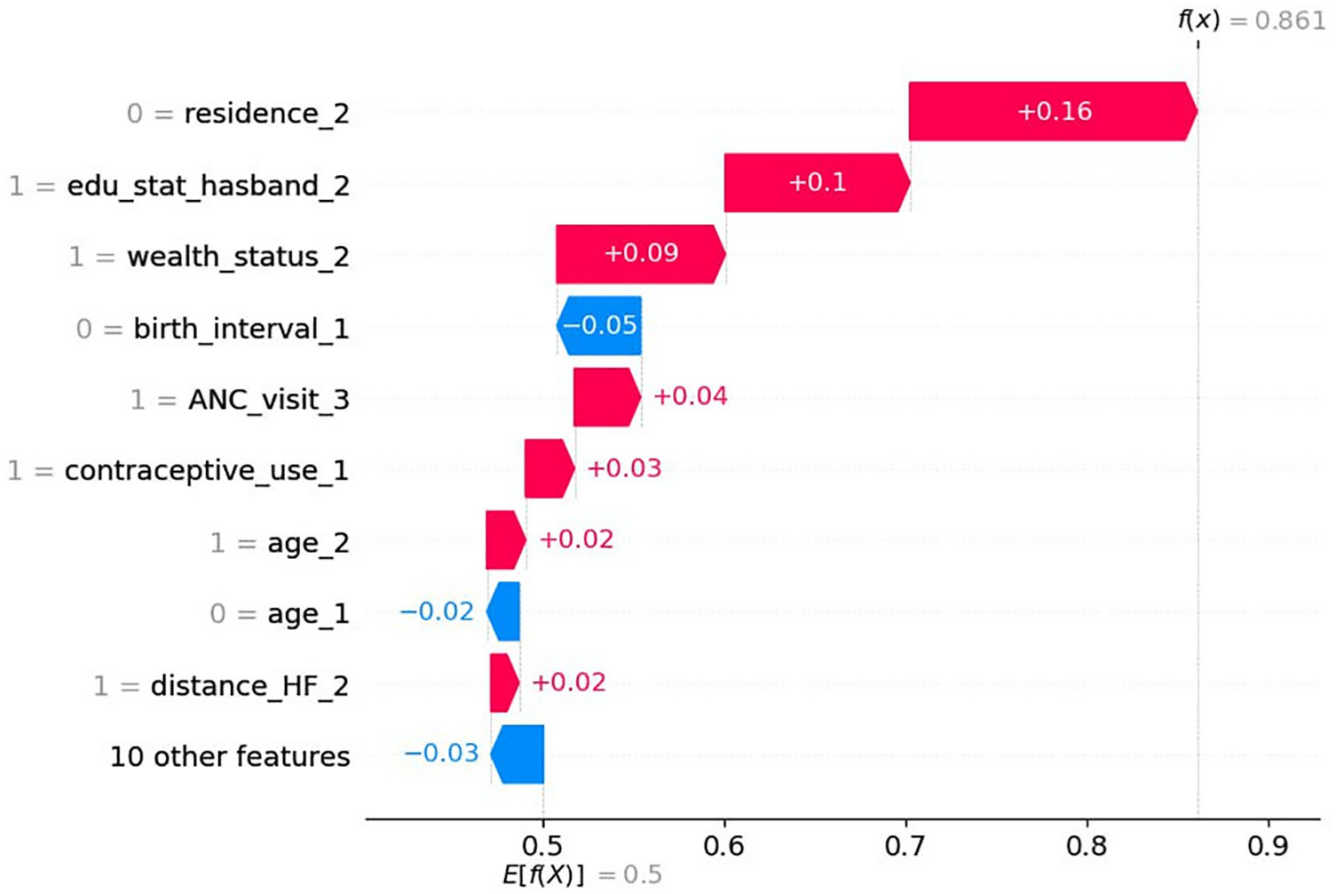
Waterfall plot displaying the prediction of the first observation.

The waterfall plots begin with the expected value of the model output on the *x*-axis {*E*[*f*(*X*)] = 0.5}, which represents the initial prediction for the given sample before considering any feature contributions. For a given observation, model outputs above this value {*E*[*f*(*X*)]} correspond to a positive class (i.e., home delivery), whereas outputs below this value correspond to a negative class (“no home delivery”). Figure 6 demonstrates how the combination of positive (in red) and negative contributions (in blue) adjusts the expected value output to a final model output [*f*(*x*) = 0.86], classified as positive class (home delivery after ANC visit).

Accordingly, not being from rural areas (0 = rural resident of women), husband’s level of secondary education (1 = secondary educational level of the husband), high household income (1 = rich household income), attending more than four ANC visits (1 = having above four ANC visits), contraceptive use (1 = contraceptive use), woman's age of 35–49 years (1 = women's age between 35 and 49), and having no difficulties with distance to health facilities (1 = having no problems of distance to health facility) increases the probability of having a home delivery after ANC visits, whereas, no short birth interval (0 = short birth interval) and woman's age not between 25 and 34 years (0 = age between 25 and 34) drives down the probability of having a home delivery after ANC visits for this particular woman (Figure 6).

## Discussion

This study aimed to assess the prediction of home delivery after ANC visits in East Africa using a supervised machine-learning algorithm. In the early stages of predictive modeling using unbalanced training data, the SVM model outperformed other classifiers. In the second stage of model prediction on balanced training data, the random forest (RF) model performed better.

The SHAP analysis based on the RF model showed that high household income, contraceptive use, short birth interval, husband’s level of primary and secondary education, residing in rural areas, attending ANC visits in the second trimester, difficulties with distance to health facilities, media exposure, and middle household income were important predictors of home delivery after ANC visits in East Africa.

Women who lived in rural areas had an increased likelihood of home delivery after ANC visits. This finding was in line with previous studies (7, 33–35). A possible explanation could be that women residing in rural areas are often far from healthcare facilities. Although services are widely accessible inside the community, transporting an ambulance in East Africa is challenging due to the region's topography and infrastructure. As a result, even though they follow the antenatal care services, women choose to deliver at home (7).

High household income decreased women's likelihood of home delivery after ANC visits. This implies that home deliveries after ANC visits were higher among women with a lower socioeconomic status compared to those who were wealthier. This finding was supported by previous studies (5–7, 11). This could be because the woman’s low income limited her from affording transport and food-related costs (36). Furthermore, it might be that most wealthy women in East Africa live in urban areas. As a result, those women have easier access to healthcare and transportation, leading to fewer home births (37).

Women who attended ANC visits in their second trimester had an increased likelihood of home delivery after ANC visits. This might be related to the level of care given throughout the ANC visit. Even with advancements over the previous 10 years, there remains a serious shortage of medical experts and unstable drug and equipment supplies (7). This result contradicts an earlier Ethiopian study that found that women who had their first ANC visit in the third trimester were less likely to give birth at home than women who had their first ANC visit in the first trimester. Thus, further study is needed to confirm this (38).

Lower levels of husband’s primary and secondary education were associated with an increased likelihood of women delivering at home after ANC visits. This finding is supported by previous studies (39–41). A possible explanation is that education is a key strategy for increasing the utilization of healthcare services; however, in the absence of universal access to education, there would be a huge disparity in the understanding of those who did and did not have difficulties in labor and delivery, as well as the risks associated with unattended deliveries (10). In addition, husbands’ knowledge improves and enables women to make decisions about where to give birth and to communicate to others (42).

Contraceptive use reduced the number of home deliveries after an ANC visit. This finding is in line with previous studies. This suggests that women who have used contraceptives will place importance on delivering in a healthcare facility. Furthermore, using contraceptives allows women to get more medical counseling, which may substantially reduce the rate of home births (7, 10, 24). Short birth intervals reduced the number of home deliveries after ANC visits. This suggests that women with short childbirth intervals were more likely to use health facilities for delivery. This could be explained by the fact that during pregnancy, women learn more about the possible risks of having frequent preterm births at ANC visits. They also help mothers develop an effective birth plan, which could reduce the possibility of the woman giving birth at home (43, 44).

Women who had no difficulties with distance to health facilities experienced a decrease in home deliveries after ANC visits. A possible explanation for this could be that, even with the lack of other options for transportation, women who live close to medical facilities are more willing to walk there. However, walking far while in labor is difficult and gets worse if labor starts at night. As a result, women choose home births (10).

Attending more than four ANC visits reduced the number of women's home deliveries after ANC visits. The number of ANC visits was also significantly associated with home delivery after ANC visits. This finding was in line with previous studies (10, 24, 45). A reason for this could be the rising number of ANC visits with medical experts, which increases the likelihood of receiving a health consultation regarding place of delivery, birth preparedness, and the value of professional birth attendants. On the other hand, women who attended fewer than four ANC visits did not communicate with medical professionals effectively and decided to deliver at home (36).

### Implications of the study

Theoretically, this study highlights the importance of sociodemographic associations in shaping maternal health outcomes. It underscores the need for multi-level interventions that address both socioeconomic factors and barriers to healthcare access. It also highlights the value of predictive modeling, particularly machine-learning algorithms, in identifying key risk factors for home delivery after ANC visits. This helps public health professionals target interventions more effectively. Overall, these findings emphasize that both healthcare system strengthening and social interventions are essential for reducing home deliveries and improving maternal and neonatal health outcomes.

Practically, this study highlights the need for targeted policy interventions to address the identified associations of home delivery. Specifically, improving access to healthcare in rural areas through mobile clinics, better transportation, and expanding healthcare facilities can significantly reduce home births. In addition, financial support and transportation subsidies are crucial to overcome wealth barriers, ensuring that women with low incomes have access to facility-based deliveries. Promoting early ANC visits through outreach programs is vital for improving maternal health outcomes and reducing home deliveries. Engaging husbands, particularly those with lower levels of education, through targeted educational campaigns can also positively impact maternal health decisions. Finally, strengthening family planning services will allow women to better plan pregnancies, ultimately reducing the likelihood of home births.

### Limitations and strengths of the study

The primary strength of this study was the use of large sample sizes and nationally representative data. The use of a sophisticated and suitable statistical method (machine-learning technique), which revealed previously undiscovered relationships and patterns in the field, was also another key point. Although machine-learning results limit the interpretation between predictors and outcome variables due to their black-box nature, this study employed the SHAP analysis to ascertain how predictors increased or decreased home delivery after ANC visits with respective predictors.

The limitation of this study was that the DHS rely on self-reported data, which may be subject to recall bias, as participants were asked to recall prior events. In addition, the cross-sectional study design limits the ability to determine causality—it only revealed relationships between factors and home births after ANC visits. Moreover, the different survey years introduce heterogeneity and future studies should consider subgroup analyses.

## Conclusion

This study found that the number of home deliveries after ANC visits was highest in Malawi, Uganda, and Kenya. The Random Forest model demonstrated the strongest predictive power of all the machine-learning models used in this study to predict home delivery after ANC visits in East Africa. The beeswarm plot of the SHAP analysis based on the RF model showed that residing in a rural area and attending ANC visits in the second trimester increases the likelihood of a home delivery after an ANC visit. In contrast, high household income, husband’s level of primary and secondary education, contraceptive use, short birth interval, having no difficulties with distance to health facilities, and attending more than four ANC visits reduced the number of women's home deliveries after ANC visits in East Africa.

As a result, this study recommends that women attend antenatal care services early and often throughout their pregnancies to ensure quality care from a qualified practitioner. In addition, education and women's empowerment are important components of programs aimed at reducing mother and infant mortality by improving the quality of health institution service utilization, especially in rural areas. Developing health facilities, promoting media health education, and encouraging women to obtain adequate information on healthcare services are essential, especially in Malawi, Uganda, and Kenya. Finally, healthcare policies should support women from low-income households.

## Data Availability

Publicly available datasets were analyzed in this study. This data can be found here: www.measuredhs.com.
