# Supplementary material for: Machine-learning algorithm to predict home delivery after antenatal care visit among reproductive age women in East Africa
Source: Front Glob Womens Health. 2025 Jun 5;6:1461475. doi: 10.3389/fgwh.2025.1461475 (PMC12176903; doi:10.3389/fgwh.2025.1461475)
Supplement: Supplementary file 1 [file Table1.docx]

# Supplementary Table 1: Variable Definitions and Categories

| Variable | Categories | Definition |
| --- | --- | --- |
| Residence | Urban, Rural | Place of residence of the mother at the time of data collection. |
| Maternal Age (Years) | 15-24, 25-34, 35-49 | Age of the mother at the time of childbirth. |
| Maternal Education | No formal education, Primary, Secondary, Higher | Highest level of education attained by the mother. |
| Marital Status | Single, Married, Widowed, Divorced | Mother's marital status at the time of data collection. |
| Wealth Index | Poor (Poorest + Poorer), Middle, Rich (Richer + Richest) | Socioeconomic status is classified into three categories for analysis. Originally measured in five categories in DHS. |
| Media Exposure | Yes, No | Whether the mother has access to mass media sources such as TV, radio, or newspapers. |
| Sex of Household Head | Male, Female | Gender of the primary household decision-maker. |
| Previous Contraceptive Use | Yes, No | Whether the mother has used any form of contraception before. |
| Timing of ANC Visit | First trimester, Second trimester, Third trimester | The trimester in which the mother started antenatal care. |
| Number of ANC Visits | One, 2-4, Above 4 | The total number of antenatal care visits during pregnancy. |
| Birth Interval | Short, Long | The time gap between consecutive births. Short = <24 months, Long = ≥24 months. |
| Husband's Education | No formal education, Primary, Secondary, Higher | The highest level of education attained by the husband. |
| Distance to Health Facility Problem | Big problem, Not a big problem | Whether the mother perceives distance as a major challenge in accessing healthcare. |
| Home Delivery after ANC Visit | Yes, No | Whether the mother gave birth at home despite attending ANC visits. |
